# In vitro evaluation of the comprehensive antimicrobial and antioxidant properties of Curtisia dentata (Burm.f) C.A. Sm: toxicological effect on the Human embryonic kidney (HEK293) and Human hepatocellular carcinoma (HepG2) cell lines

**DOI:** 10.17179/excli2015-351

**Published:** 2015-08-24

**Authors:** VO Fadipe, NI Mongalo, AR Opoku

**Affiliations:** 1 Department of Chemistry, University of Zululand, Private Bag X1001, KwaDlangezwa, 3886, South Africa; 2 College of Agriculture and Environmental Sciences (CAES) Laboratories, University of South Africa, Private Bag X6, Florida, South Africa; 3 Department of Biochemistry and Microbiology, University of Zululand, Private Bag X1001, KwaDlangezwa, 3886, South Africa

**Keywords:** Curtisia dentata, ß-sitosterol, ethnomedicine, cytotoxicity, antioxidant, antimicrobial

## Abstract

*Curtisia dentata* is used in African traditional medicine to treat variety of infections. *C. dentata* leaves were collected from Buffelskloof Nature Reserve, South Africa. The ethanol, chloroform, ethyl acetate and acetone extracts were evaluated for antimicrobial activity using micro dilution assay against *Escherichia coli*, *Pseudomonas aeruginosa*, *Mycobacterium smegmatis, Mycoplasma hominis, Candida albicans* and some clinical isolates of *Moraxella catarrhalis*, *Proteus mirabilis* and *Staphylococcus aureus* isolated from HIV patient. Acetone extract exhibited lowest MIC of 0.01 mg/ml against *Candida albicans* compared to other extracts. Besides lupeol, betulinic acid and ursolic acid, β-sitosterol was isolated for the first time from *C. dentata* leaves and exhibited antimicrobial activity with MIC values ranging from 0.20 to 6.25 mg/ml. Furthermore, the ethanol extract and the four isolated compounds revealed microbicidal effect, with MIC index of less than 4. Ethanol extract revealed the best total activity of 2400 ml/g against *Mycoplasma hominis*. Cytotoxicity of the isolated compounds was further investigated against the Human embryonic kidney (HEK293) and Human hepatocellular carcinoma (HepG2) cell lines using the MTT assay. Ursolic acid exhibited the lowest LD_50 _of 122.4 µg/ml against HEK293 cell line while lupeol exhibited LD_50 _of 278.8 and 289.4 µg/ml against HEK293 and HepG2 respectively. Lupeol exhibited low selectivity index. Ethyl acetate and acetone extracts were further investigated for antioxidant activity against 2,2-diphenyl-1-picrylhydrazyl (DPPH). The acetone extract exhibited potent inhibition of DPPH compared to ethyl acetate extract. The findings of the current work validate the use of the plant species in the treatment of various human infections.

## Introduction

Microbial resistance to common antibiotics is becoming a norm and an enormous threat to general health care facilities, especially in poorer countries with little or improper medical facilities and resources (Marasini et al., 2015[18]; Chovanová et al., 2013[5]; Vigneshwari et al., 2014[49]). The situation is worsened by increase in HIV-AIDS infections, tuberculosis and the defaulting patients in hospitals. Although medicinal plants serves as an alternative medicine to most rural communities, mode of action and their general safety remains unknown. Although pharmacological testing and research on natural products represent a major strategy for discovering and developing new drugs (Priyadharshini and Sujatha, 2013[37]; Nasri and Shirizad, 2013[27]), the cytotoxicity of major plant derived indigenous products used in most remote areas remains a challenge and risk to health of many rural people. The free radicals are generated by exogenous reactions and respiration in the human body. The reactive oxygen species (ROS) play a role in the development of various devastating ailments which includes cancer, arthritis, asthma, dementia, mongolism, carcinoma and others (Narayanaswamy and Balakrishnan, 2011[26]). However, some plant extracts and other plant derived materials and products have been implicated in quenching of those free radicals, thereby delaying or curing some illnesses (Mongalo et al., 2015[20]; Mosa et al., 2011[22]).

Generally* Curtisia *species are woody, leathery simple leaves with revolute margins, opposite phyllotaxy, lacking stipules, hermaphroditic flowers, inferior ovary and possess copious endosperm (Yembaturova et al., 2009[50]). *Curtisia dentata* is traditionally used in South African traditional medicine to treat stomach ache, diarrhoea, sexually transmitted infections, aphrodisiac, as blood purifier and as a purgative (Cunningham, 1988[6]; Hutchings et al., 1996[15]). Geographically, the species is restricted to Southern Africa, mostly long the southern and eastern coasts of South Africa and extends into eastern Zimbabwe, Mozambique and Swaziland (Yembaturova et al., 2009[50]). Besides being threatened by bark harvesting for the medicinal plant trade (Ndawonde, 2006[28]), the leaves of the species have been recently found to contain important pentacyclic triterpene compounds such as betulinic acid, ursolic acid, 2α-hydroxyursolic acid and lupeol (Fadipe et al., 2014[11]; Shai et al., 2008[40]). 

Besides exhibiting lipid peroxidase and ferric oxide activity, the hydro-alcoholic extract of the stem bark exhibited moderate free radical scavenging activity against both ABTS and DPPH (Oyedemi et al., 2012[35]). The current paper is aimed at investigating the antimicrobial activity of various leaves extracts of *Curtisia dentata* and the isolated compounds, including β-sitosterol which was isolated for the first time, against opportunistic organisms, mostly isolated from HIV-AIDS patient. The paper further explores the cytotoxity properties against human cell lines and the free radical scavenging activity of the plant species against a known stable free radical.

## Materials and Methods

### Collection and storage of plant materials

*Curtisia dentata* leaves were collected from the Buffelskloof Nature Reserve situated in Mpumalanga Province, South Africa. The plant was authenticated and identified by Mr John Burrows, Botanist and Reserve Manager, Buffelskloof Private Nature Reserve and a voucher (specimen No: B.C.Turpin-2062) has been deposited in the Herbarium of the Buffelskloof Private Nature Reserve. The leaves were then washed with distilled water, dried on a laboratory bench for five weeks and ground into thin powder (2 mm mesh) using Scientec Hammer Mill, Germany. Powders were kept in plastic bottles at room temperature until required.

### Chemicals and reagents

Acetone, chloroform, ethyl acetate, ethanol (all were AR grade from Merck, South Africa), DPPH, (Sigma, Germany), Muller Hinton broth, Muller Hinton agar (Oxoid) ADC Middlebrook supplement, Middlebrook 7H9 broth (Fluka), glycerol, Iodo-nitro-terazolium chloride (Fluka), Mycoplasma agar, Mycoplasma broth, Yeast malt broth and Yeast Malt agar (Oxoid).

### Extractions

About 10 g of the ground leaves were extracted with 40 ml acetone, ethanol, chloroform and ethyl acetate three times separately using a sonicator at 15 °C. The extracts were then filtered through Whatman's No.1 paper and then concentrated using Buchi Rotary evaporator. The residues were then weighed and kept in a refrigerator until needed.

### Antimicrobial activity

#### Minimum Inhibitory concentrations (MIC) assay

The micro plate broth dilution assay was used to assess the minimal inhibitory concentration (Eloff, 1998[9]) with slight modification. The 12 hour old culture was diluted 1:100 with freshly prepared Muller-Hinton broth. About 100 µl of extracts (50 mg/ml in 5 % respective original solvent) were added to multi well plate containing 100 µl of freshly prepared broth and serially diluted. Plates were then incubated over night at 37 °C. About 40 µl of 2 mg/ml freshly prepared iodo-nitro-tetrazolium chloride were added to each well and incubated for 1 hour at the same temperature. For *M. hominis*, Mycoplama CM0403 broth (Oxoid) was supplemented with mycoplasma supplement G, while *M. smegmatis* was grown on Middlebrook 7H9 broth (Fluka) supplemented with glycerol and ADC middlebrook growth supplement with an incubation time of 48 hrs. *C. albicans* was maintained on Yeast malt agar, grown on yeast malt broth and similar procedure was used as in bacterial strains and the plates were incubated for 12 hrs. The MIC was defined as the lowest concentration of the extract to inhibit bacterial growth. 

#### Bactericidal/Fungicidal Concentrations (MBC/MFC), total activity and MIC index

Shortly, a loopful of the microorganism in the wells showing little or no growth in the MIC assay were selected and sub-cultured on the petri plates containing an agar for different microbes. The MBC or MFC were defined as the lowest concentration that showed no bacterial or fungal growth in the subcultures (N'guessan et al., 2007[30]). To compare the activity of various plant extracts, the total activity in mL/g was calculated by dividing the total mass in mg extracted from 1 g of the plant material by the MIC value in mg/ml (Eloff, 2000[8]), while the MIC index was calculated by dividing the MBC in mg/ml with the MIC exhibited by the same extract in mg/ml (Fernandez et al., 2012[14]). The MIC index of ≤ 4 was defined as exhibiting bactericidal/fungicidal effect, > 4 as bacteriostatic effect while ≥ 16 as ineffective (Stefanovic and Comic, 2011[44]; Okeleye et al., 2013[33]).

### Antioxidant activity

#### DPPH free radical scavenging activity

DPPH scavenging activity of the methanol extract of the plant was carried out according to method previously described (Opoku et al., 2002[34]). Decolourisation of DPPH (purple) upon addition of the extract indicated radical scavenging activity and this was measured after 30-60 min at 517 nm. Percentage of inhibition was calculated as 

% Scavenging Inhibition = [1-A_t_/A_0_] X 100, and the results were reported as mean ± SE (n = 3).

#### Isolation of ß-sitosterol from Curtisia dentata leaves

Dried clean ethanol extract (8 g) was subjected to column chromatograph (40.5 X 530.5 mm) using silica gel 60(180 g, 0.04 -0.063 mm; 230-400 mesh) supplied by Merck (Darmstadt, Germany). The clean ethanol extracts were chromatographed using gradient elution of hexane-ethyl acetate in a 10 % increase and collecting 80 mL fractions. Twenty-five (25) fractions were collected and monitored based on their TLC (F254-Merck, Whitehouse Station, NJ, USA) by visualization was achieved by UV light (254 nm) and spray with 20% H_2_SO_4_ in MeOH followed by heating in the oven (105 °C ). Compound I (89.34 mg) obtained from fractions 7-11 as single spot. The compounds 2, 3 and 4 were isolated and reported before (Fadipe et al., 2014[11]). 

### Spetroscopic analysis

#### Infra-red (IR)

The infra-Red (IR) spectroscopy determination was carried out using Perkin Elmer Spectrum 100 FTIR spectrometer. 

#### Nuclear magnetic resonance (NMR)

^1^H, ^13^C NMR and all 2D spectra were recorded on a Bruker Avance instrument operating at 400 MHz, Chemical shifts are reported as δ values (ppm) relative to an internal standard of tetramethylsilane (TMS) or to the solvent line of CDCl3 (^δ^H = 7.26 ppm, ^δ^C = 77.16 ppm). 

#### High-resolution-mass spectroscopy (HR-MS)

High-resolution mass data were obtained using a Bruker micro TOF-Q II ESI instrument operating at ambient temperature.

#### Melting point (mp)

Melting points of the compounds were determined on a Stuart Scientific SMP3 apparatus.

#### Compound 1

IR (cm-1): 3377.8 (OH group), 2921.4 (aliphatic C-H), 1645.7 (olefinic carbon at [C-5]), 1382.1 (geminal dimethyl group). 1H-NMR (CDCl3) δ (ppm) 3.2 (1H, *m*, H-3), 5.1(1H, *t*, H-6), 0.8 (3H, *s*, Me-18), 1.6 (3H, *s*, Me-19), 1.2 (3H, *d*, *J *=6.5 Hz,Me-21), 1.06 (3H, *d*, *J *=6.7 Hz, Me-26), 0.9 (3H, *d*, *J *=6.7 Hz,Me-27), 1.1 (3H, *t*, *J*=7.4 Hz, Me-29).13C-NMR (CDCl3) δ (ppm) 38.85 (C-1), 31.19 (C-2), 79.1 (C-3), 41.7 (C-4), 124.5 (C-5),121.8 (C-6), 32.7 (C-7), 33.4 (C-8), 47.7 (C-9), 37.2 (C-10), 23.78 (C-11), 39.7 (C-12), 46.9 (C-13), 59.1 (C-14), 26.6 (C-15), 28.1(C-16), 55.2 (C-17), 15.7 (C-18), 17.9 (C-19), 38.6 (C-20), 17.4(C-21), 34.8 (C-22), 27.34 (C-23), 47.3 (C-24), 29.7 (C-25), 18.4(C-26), 21.5 (C-27), 26.2 (C-28), 16.8 (C-29).

### Cytotoxicity studies

Shortly, the cytotoxicity studies were carried out using MTT Cell Proliferation Assay as described by Mosman (1983[23]). The Human embryonic kidney (HEK293) and Human hepatocellular carcinoma (HepG2) cells were all grown to confluences in 25 cm^3^ flasks. This was then trypsinized and plated into 96 well plates at specific seeding densities (5x10^4^ cells per well). Cells were incubated overnight at 37 °C. Medium was then removed and fresh medium (MEM + Glutmax + antibiotics) was added. Isolated compounds (50-300 μg/ml) were then added in triplicate and incubated for 4 h. Antibiotics used were 100 µg/ml of both pencicllin G and streptomycin. Thereafter medium was removed and replaced by complete medium (MEM + Glutmax + antibiotics + 10 % Fetal bovine serum). After 48 h cells were subjected to the MTT assay and the results for different concentrations were reported as mean±SE, using Microplate reader (Meter tech. Σ 960, USA) at 570 nm. The wells with cells only were used as control. The percentages of inhibition were then calculated using 

Percentage cell inhibition = 100 -Abs (Sample) / Abs (Control) x 100 

(Sreejaya and Santhy, 2013[43]), while the IC_50_ were obtained from the logarithmic curve of % inhibition v/s concentrations of the isolated compounds. The Selectivity index was calculated as follows: SI=LD_50_ in mg/ml/ MIC in mg/ml (Bagla et al., 2014[1]).

## Results and Discussion

### Antimicrobial activity

The microbial resistance of various strains against common antibiotics, especially in developing countries, has resulted in tremendous selective pressure on antibiotics. These have prompted the need to investigate the antimicrobial potential of traditional medicinal plants against pathogenic strains of both bacterial and fungal strains. A variety of extracts, essential oils and compounds from South African medicinal plants have been investigated for antimicrobial activity against variety of etiologic agents of devastating human infections (Buwa and Van Staden, 2006[3] ; Tshikalange et al., 2008[47]; van Vuuren, 2008[48]; Mulaudzi et al., 2015[25]) with the intention of finding new and alternative medicines. 

The antimicrobial properties of *Curtisia dentata* leaf extracts and isolated compounds are presented in Table 1(Tab. 1). The acetone extract exhibited lowest MIC of 0.01 mg/ml against *Candida albicans* compared to other extracts while ethanol and chloroform extracts exhibited MIC of 0.10 mg/ml against *Mycoplasma hominis*. Contrarily, Shai et al. (2009[39]), reported MIC values of leaves and stem bark acetone extracts of 0.11 and 0.61 mg/ml respectively. Recently, Nielsen et al. (2012[31]), reported the MIC values of the methanol extracts of the stem bark of 156.25 µg/ml against β-lactamase *Escherichia coli*, ampicillin-resitant *Klebsiella* *pneumoniae*, chloramphenicol resistant *Citrobacter, *methicilin-resistant* Staphylococcus aureus *(MRSA) and carbennicillin-resistant *Pseudomonas* *aeruginosa *(CRCF), while the leaves exhibited MIC values of 78.12 and 625 µg/ml against MRSA and CRCF respectively. Although these results are not comparable to the data presented in the current paper due to differences in type and origin of the microbial strains used and other environmental conditions, including collection times, they may well suggest that most of the biological active secondary metabolites may be embedded into the leaves than the stem bark.

The ethyl acetate and ethanol extracts exhibited potent MBC of 0.20 against *Mycoplasma hominis* while chloroform extract exhibited similar MBC against *C. albicans*. According to Chitemerere and Mukanganyama (2011[4]), MIC values of 0.06 to 0.5 mg/ml are referred to as potent when using similar method as the one in our study. Earlier, the diethyl ether extract exhibited moderate MIC of 3.13 mg/ml against *Proteus mirabilis* (Fadipe et al., 2014[11], 2015[12]). Betulinic acid revealed most potent antimicrobial activity compared to other isolated compounds. In other studies, betulinic acid isolated from different plants was found to possess variety of biological activity, including anti-HIV, anti-inflammatory, anti-malarial, anti-feedant, anti-parasitic, catalytic activity of Topo II, anticancer and actinociceptive activity (Theo et al., 2009[45], Moghaddam et al., 2012[19]).

Interestingly, the organic extracts from *C. dentata* revealed most potent MIC values against common pathogenic sexually transmitted infecting organisms and those isolated from HIV patient in our current study. These results in a way, validates the use of the plant species in the treatment of such infections. Besides being recognised as the genital and neonatal pathogen, *M. hominis* may inhabit the immune systems of both immune-compromised and immune-competent individuals, resulting in serious extra-genital infections such as pneumonia, mediastinitis, pericarditis, endocarditis, osteitis, arthritis and wound infections (Pascual et al., 2010[36]). Moreover it may cohabit with other organisms which may include *C. albicans *which causes vaginitis (Njunda et al., 2011[32]). Besides exhibiting bactericidal/fungicidal activity, the ethanol extract exhibited the best total activity of 2400 mL/g, suggesting that one gram may be diluted with 2400 ml of a solvent and still inhibit the growth of the organism. Interestingly, bactericidal antibiotics are recommended in the treatment of severely ill and immunosuppressed patients (Nemeth et al., 2015[29]). Although the results obtained in the current work validates the use of the plant in the treatment of variety of infections, the mode of action of these extracts and some isolated compounds remains unknown. Although both the extracts and isolated compounds exhibited potent antimicrobial activity against *Candida albicans* and *Mycoplasma* *hominis*, which are implicated as causative agents of sexually transmitted infections (STIs), there is a need to explore the biological activity of the plant species against other organisms belonging to the similar sphere like *Neisseria gonorrhoea*, *Trichomonas vaginalis* and other organisms causing several related infections.

### Cytotoxicity studies

According to Doughari et al. (2011[7]), the rigorous toxicity studies of *C. dentata *needs to be carried out to enable identification of new biologically active compounds and cautions to be issued on dangerous practices or toxicity effects, if any, as the species is extensively used in the treatment various human infections. The results for the cytotoxicity investigations are shown below (Table 2(Tab. 2)) and suggest that the isolated compounds inhibit the growth of selected human cells in a dose dependent manner. Earlier, the acetone and dichloromethane extracts of *C. dentata* reportedly exhibited LC_50_ of 0.007 and 0.024 µg/ml respectively in an MTT assay against Monkey kidney cells (Shai et al., 2008[41]). The higher LD_50_ implies that it would take a large quantity of the extract to cause a toxic response, while small LD_50_ values are highly toxic and could be dangerous (Okeleye et al., 2013[33]). In our current study, both betulinic acid and β-sitosterol exhibited LD_50_ of > 300 µg/ml against the selected cell lines, suggesting that the compounds are less toxic. Ursolic acid exhibited the lowest LD_50 _of 122.4 µg/ml against HEK 293 cell line while lupeol exhibited LD_50 _of 278.8 and 289.4 4 µg/ml against HEK 293 and Hep G2 respectively. However, according to Sahranavard et al. (2009[38]), the isolated compounds are referred to as toxic when exhibiting LD_50 _of about 100 µg/ml or less. Some isolated compounds and plant extracts from plant materials reportedly exhibited very low IC_50_, hence toxic (Mthethwa et al., 2014[24]; Behzad et al., 2014[2]; Tiwary et al., 2015[46]).

The β-sitosterol and betulinic acid revealed percentage inhibition of 25.0 to 41. 30 %. According to El-Sharkawy et al. (2013[10]), the percentage of inhibition ranging from 25 to 50 is referred to as normal. Elsewhere, β-sitosterol exhibited LD50 of 21.9 and 21.6 against normal (HFB4) and cancer (MCF7) cells respectively (Singab et al., 2012[42]). Generally, the isolated compounds are less toxic hence relevant to use in pharmacological setups.

### Selectivity index

The selectivity index (SI) of ursolic acid and lupeol against HEK 293 and HepG2 are reported in Table 3(Tab. 3). SI indicates the cytotoxic selectivity or safety of the crude extract or isolated compound against the selected cell lines (Machana et al., 2011[16]). The compounds exhibited low selectivity index values, indicating the similar cytotoxicity and antimicrobial activity. According to Makhafola et al. (2014[17]), the *in-vitro* toxicity does not equate to in vivo toxicity because of difference in physiological microenvironment in live animal and tissue culture. Moreover, other factors relating to chemical kinetics which may include absorption, biotransformation, distribution and excretion, which influence the exposure at the level of target cells in vivo, cannot be adequately simulated *in vitro*. 

### Antioxidant activity

The results for the antioxidant activity of both acetone and ethyl acetate are shown in Table 4(Tab. 4). The acetone extract exhibited better inhibition of DPPH than ethyl acetate extract, while ascorbic acid completely inhibits DPPH at lower concentrations. Acetone extract inhibits DPPH at a comparable state at 0.08 mg/100 ml. According to Mongalo et al. (2012[21]), the extract is a good inhibitor of DPPH if it gives inhibition of about 75 % at 0.75 mg/100ml, suggesting that the acetone extract of the plant species in the current study exhibited moderate free radical scavenging activity while ethyl acetate was relatively poor. 

However, there is a need to explore the antioxidant properties of the extracts against other forms of radicals. The antioxidant activity may be of greater important in preventing oxidative stress which may be involved in many fatal infections and diseases.

### Isolated compounds

**Figure 1:**
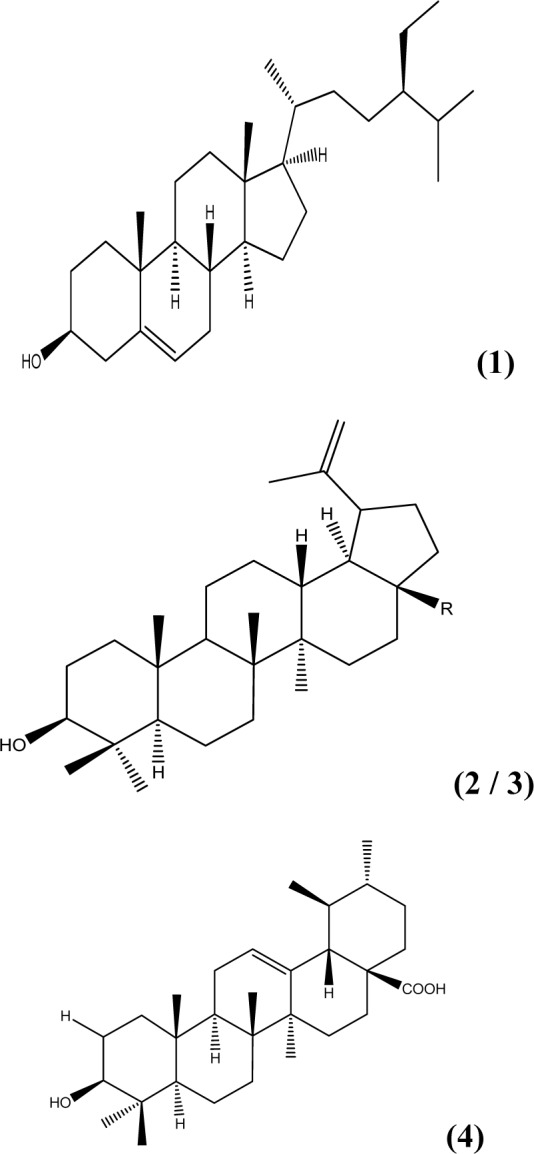
Isolated compounds: (1) β-Sitosterol, (2) Lupeol, (3) R=CH_3_, Betulinic acid - R=COOH, (4) Ursolic acid

The newly isolated compound (**1**) was isolated and identified as β-Sitosterol (NMR data not shown), while the other three compounds (**2**, **3** and **4**) were identified earlier (Fadipe et al., 2014[11]) (Figure 1(Fig. 1)).

## Conclusions

*Curtisia dentata* leaves extracts exhibited good antimicrobial activity against the selected microbial strains. The activity exhibited by ethanol extract further resulted in the isolation of compounds such as lupeol, betulinic acid ursolic acid and the newly isolated compound known as β-sitosterol which also exhibited potent antimicrobial activity. The isolated compounds were less toxic to HepG and HEK 293 cell lines. The acetone extract exhibited better DPPH free radical scavenging activity compared to the ethyl acetate extract. However, there is a need to explore the antioxidant activity of these extracts using other assays. The current study validates the use of the plant species in the treatment of various human infections, including sexually transmitted infections. However, there is still a need to investigate the antimicrobial activity of these extracts against other pathogenic organisms.

## Future Studies

We are currently assessing the anti-mycobacterial activity of the isolated compounds individually and in combination. We also need to explore the antimicrobial effect of the aqueous extracts of the plant species against pathogenic human strains. We are also investigating the phyto-chemistry of the acetone extract as it revealed activity against selected microbes.

## Acknowledgements

Authors are deeply thankful to the District Hospital (Kwa-Nongoma area) for the generous donation of microorganisms. We are also deeply thankful to National Botanical Institute (Nelspruit) for allowing us to collect the leaves of the plant species used in the current study.

## Conflict of interest

The authors declare no conflict of interest.

## Figures and Tables

**Table 1:**
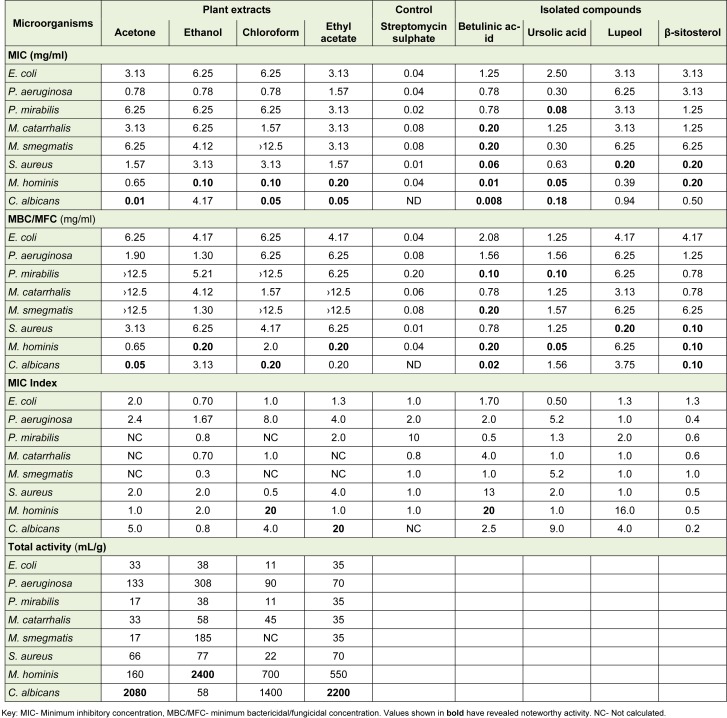
Antimicrobial activity of *Curtisia dentata* leaf extracts and isolated compounds

**Table 2:**
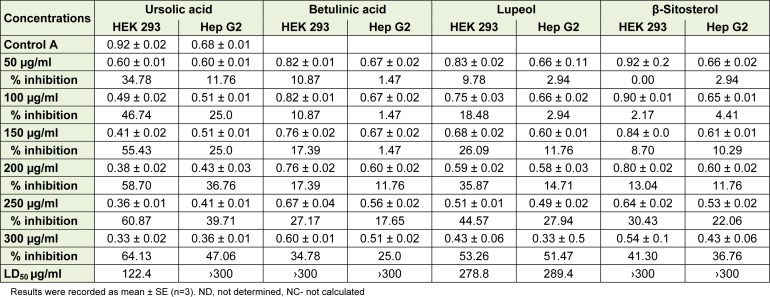
Cytotoxicity studies of the isolated compounds from *Curtisia dentata* leaves

**Table 3:**
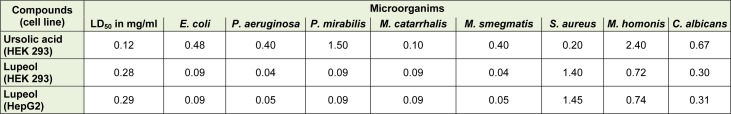
Selectivity index of isolated compounds in mg/ml

**Table 4:**
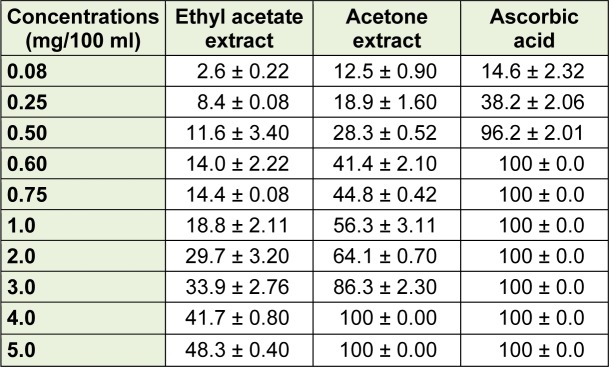
Antioxidant activity of extracts from *Curtisia dentata* leaves
